# Does Coherence Affect the Multielectron Oxygen Reduction Reaction?

**DOI:** 10.1021/acs.jpclett.3c02594

**Published:** 2023-10-12

**Authors:** Anu Gupta, Anil Kumar, Deb Kumar Bhowmick, Claudio Fontanesi, Yossi Paltiel, Jonas Fransson, Ron Naaman

**Affiliations:** † Department of Chemical and Biological Physics, Weizmann Institute of Science, Rehovot 7610001, Israel; ‡ Department di Ingegneria, DIEF, MO26, University of Modena, 41125 Modena, Italy; § Department of Applied Physics and Center for Nanoscience and Nanotechnology, The Hebrew University, Jerusalem 9190401, Israel; ∥ Department of Physics and Astronomy, Uppsala University, Uppsala 752 36, Sweden

## Abstract

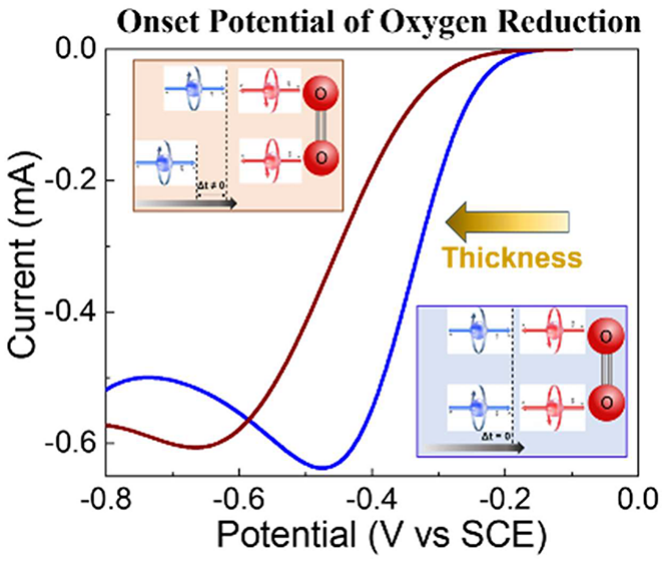

The oxygen reduction reaction (ORR) is the key for oxygen-based respiration and the operation of fuel cells. It involves the transmission of two pairs of electrons. We probed what type of interaction between the electrons is required to enable their efficient transfer into the oxygen. We show experimentally that the transfer of the electrons is controlled by the “hidden property” and present a theoretical model suggesting that it is related to coherent phase relations between the two electrons. Using spin polarization electrochemical measurements, with electrodes coated with different thicknesses of chiral coating, we confirm the special relation between the electrons. This relation is destroyed by multiple scattering events that result in the formation of hydrogen peroxide, which indicates a reduction in the ORR efficiency. Another indication for the possible role of coherence is the fluctuations in the reaction efficiency as a function of thickness of the chiral coated electrode.

Many molecular reactions involve more than a single electron transfer process. Often the injection or removal of subsequent electrons from an already partially reduced or oxidized system requires overcoming an electrostatic barrier. Regarding reduction, it results from the repulsion between the partially reduced system and the additional electron, whereas for oxidation, it results from the Coulomb attraction between the partially oxidized species and the electron that must be removed. However, sometimes the injection or removal of the second electron has a very low barrier, and these processes may even be favorable in a situation called “potential inversion”. Namely, the two-electron process is more favorable energetically than the single electron one.^1^ Often it is assumed that in these cases, following the transfer of the first electron, the system undergoes a structural change that enables the transfer of the second electron. However, this explanation cannot be valid for cases in which the single- and the two-electron processes result in different products, due to spin restriction, for example. Hence, in such cases the two electrons are expected to be transferred simultaneously.^2^ The question we address here is what the relationships are between the two electrons that are transferred “simultaneously”. We aim to address this question in relation to the oxygen reduction reaction (ORR).

It is well established that in ORR, four electrons are transferred as two pairs.^3−5^ The reaction is spin forbidden because the oxygen ground state is a triplet, whereas the products are all singlets. We show here that the interaction between the two electrons in each pair determines the electrochemical potential required for the reaction and affects the reaction mechanism. The ORR results in water as the lower energy product and hydrogen peroxide as another product that is higher in energy.^6^ However, it was found that the water production, itself, has a high barrier due to the need to overcome the spin restriction in the reaction.^7^ In a recent study, it was demonstrated that by having the two electrons in the same spin state the reaction rate is enhanced, since the spin restriction is removed and the reaction can occur on a triplet potential energy surface to produce water. However, if the spins are not coaligned, the hydrogen peroxide production is enhanced.^8^ When adding various gases, which can affect the spin alignment of the transferred electrons, the reaction rate for direct reduction efficiency is reduced, whereas the formation of hydrogen peroxide is enhanced.^9^

Here we propose that in addition to spin control the efficiency of the ORR depends on the two electrons in the pair being coupled. The requirement for coupling was established both experimentally and by model calculations. Although experimentally it is very difficult to verify if this coupling relates to a coherent relation, we suggest that since the two-electron transfer to the oxygen occurs on a subnanometer scale, it is reasonable to assume that the coupled electrons maintain coherency and a phase relationship between them even under ambient conditions. The notion that there must be a phase relationship between the two electrons in a “two-electron process” was suggested theoretically before using a classical model,^10^ and is shown here in the quantum mechanical model.

To determine the importance of the interaction between the electrons, we first investigated the electrochemical ORR with either a magnetic working electrode or a working electrode coated with a chiral polymer film of various thicknesses. The chiral polymer was prepared by spin dependent electropolymerization of achiral 2-vinylpyridine, as described before.^11^ The thickness of the film depends on the time of electropolymerization; it was calibrated using AFM. The spin-dependent current through the films, as a function of their thickness, was determined by magnetic contact AFM, mc-AFM (Figure 1A). By varying the magnetic orientation of the magnetic substrate, current was measured for each spin direction and for 7 samples of different thicknesses, when the north pole of the magnet points either toward the adsorbed layer, *I*_up_, or away from it, *I*_down_, and the potential was set to 3 V (Figure 1B). The ratio between the current of the different spins increases with increasing thickness. At a thickness of 4 nm, the ratio is about 3:2, whereas at 20 nm it is 2:1. Hence, the percentage of spin polarization (blue curve), SP = , increases gradually and monotonically, whereas the current decreases. Figure 1C presents the difference between the currents when the magnetic direction of the substrate, from which the electrons are injected, is switched. The figure illustrates that the absolute magnitude of the spin-polarized current decays with increasing thickness, despite an increase in the spin polarization.

**Figure 1:**
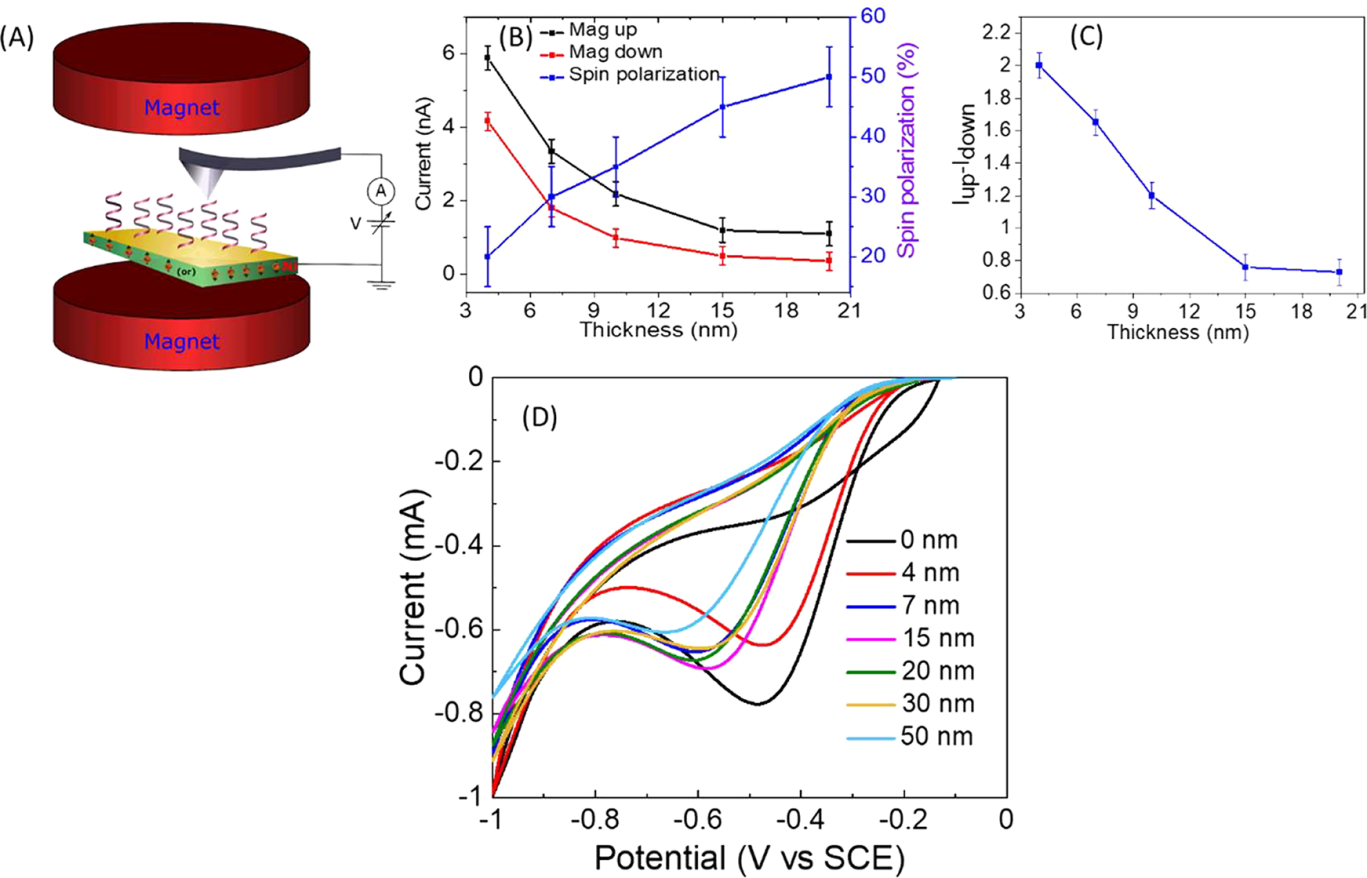
(A) The magnetic contact AFM (mc-AFM) setup. (B) The current through the polymer layer, as a function of the layer thickness, measured by mc-AFM when the potential is 3 V, with the magnetic north pole of the substrate pointing toward (up) or away (down) from the polymer film (black and red lines, respectively). The blue line denotes the spin polarization, SP =  when *I*_up_ and *I*_down_ are the current with the magnet north pole pointing up or down, respectively. (C) The difference in the currents for the two orientations of the magnetic field, *I*_up_ – *I*_down_, as a function of the polymer thickness. (D) The current versus voltage (CV) curves obtained for working electrodes coated with various thicknesses of polymer. Zero thickness refers to a magnetic electrode whose magnetic field points up and has no polymer coating.

The ORR was investigated by using electrochemistry with a three-electrode cell configuration. The reference electrode was an Hg/Hg_2_Cl_2_/saturated KCl (saturated calomel electrode, SCE), and the counter electrode was a Pt wire. A 0.1 M KOH solution was used as the electrolyte at pH = 12.6. The working electrode was fixed to the bottom of a Teflon cell through an O-ring having an area of 0.76 cm^2^. Current versus voltage, CV, curves were obtained for either a magnetic gold-coated Ni working electrode that was magnetized out of plane (zero thickness) or with the gold electrode coated with a different thickness of chiral polymer or with monolayers of DNA or oligopeptides.

*Chiral Polymer*. Figure 1D presents the CV curves obtained for the polymer-coated working electrode, and Table 1 summarizes the parameters of each curve. The efficiency of the reaction can be evaluated from the threshold potential of the curve, the peak potential, and the current.

**Table 1:** Parameters Obtained from the ORR Electrochemical Studies for an Electrode Coated with a Chiral Polymer of Different Thicknesses. The Onset Potential Is Defined as the Potential at a Current of 0.1 mA

polymer thickness (nm)	peak current density (mA/cm^2^)	current density at −0.5 V (mA/cm^2^)	peak potential (−V)	onset potential (−V)
0	1.00 ± 0.02	1.00 ± 0.02	0.49 ± 0.01	0.25 ± 0.03
4	0.80 ± 0.02	0.78 ± 0.02	0.47 ± 0.01	0.26 ± 0.04
7	0.83 ± 0.02	0.72 ± 0.01	0.59 ± 0.01	0.33 ± 0.05
15	0.87 ± 0.02	0.76 ± 0.02	0.57 ± 0.00	0.32 ± 0.02
20	0.84 ± 0.01	0.73 ± 0.02	0.58 ± 0.02	0.32 ± 0.05
30	0.83 ± 0.01	0.74 ± 0.01	0.58 ± 0.01	0.32 ± 0.02
50	0.76 ± 0.01	0.54 ± 0.01	0.65 ± 0.01	0.35 ± 0.01

As the film on the electrode becomes thicker, both the threshold potential and peak potential shift to higher values, indicating a higher barrier for the reaction. Since the ORR reaction is more efficient for spin-polarized electrons, one would expect that the reaction efficiency would decrease slowly with the thickness of the chiral polymer, namely, with the decrease in the current of the spin-preferred electrons (the black curve in Figure 1B). However, when the important parameters of the CV plots are presented versus the film thickness (Figure 2 and Table 1), they indicate an abrupt drop in the reaction yield following a thickness of about 7 nm of the polymer film.

**Figure 2:**
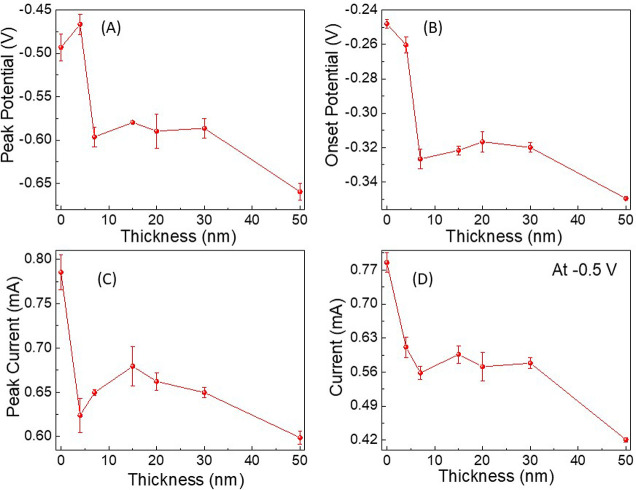
Dependence of the characteristic parameters of the CV curves on the thickness of the chiral polymer film. (A) The dependence of the peak potential. (B) The onset potential. (C) The current at the peak of the curve. (D) The current at −0.5 V.

Another interesting observation is the crossing in the CV curve, observed for zero polymer coating or when the electrode is coated with a 4 nm polymer (Figure 1D). This crossing indicates that the reduction product reacts further and therefore the CV process is not completely reversible.^12,13^

Figure 2A presents the current obtained at the peak of the reduction current as a function of film thickness. It shows that the reaction rate decreases sharply for polymer films with a thickness above about 7 nm. Similar results are obtained for the onset potential (Figure 2B), for the current at the peak of the CV (Figure 2C), and for the current at −0.5 V, which is near the peak of the CV for the bare electrode (Figure 2D). Table 1 summarizes all of these relevant parameters.

As hydrogen peroxide production is known to be a byproduct when the ORR is not efficient,^8^ we investigated its production as a function of the thickness of the chiral polymer layer deposited on the working electrode. The electrolyte solution was exposed to current for 30 min when the potential in the electrochemical cell was set to −0.5 V (Figure S1(A)). The electrolyte was then removed, and the hydrogen peroxide concentration was monitored by the addition of o-tolidine as a redox indicator.^14,15^ In the presence of H_2_O_2_, a yellow absorption peak appears at about 436 nm (see Figure S1(B)). This peak characterizes the complete two-electron oxidation product of o-tolidine formed by the reaction with hydrogen peroxide.^16^ The intensity of the absorption peak, divided by the current in the electrochemical cell, as a function of the film thickness is presented in Figure 3. The results presented account for the reduced current when the film thickness increases. As shown in Figure 3, indeed, the reduction in the efficiency of the ORR reaction is accompanied by an increase in the production of hydrogen peroxide. The hydrogen production increases abruptly with increased polymer thickness to above about 7 nm; the same thickness at which the reaction rate drops, as indicated in Figure 2. This increase in hydrogen peroxide concentration does not result from the change in the current since the signal is normalized to the current.

**Figure 3:**
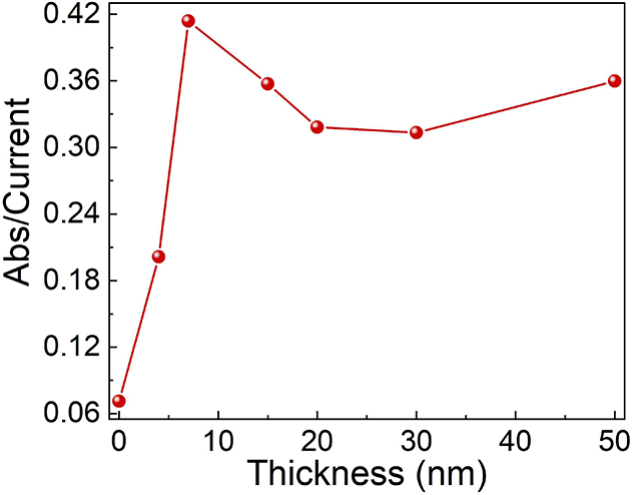
Hydrogen peroxide production in ORR as a function of the thickness of the chiral polymer film coating the working electrode. The signal is normalized to the current at −0.5 V.

*DNA*. In the past it was realized in several studies that the conduction through double stranded DNA^17,18^ and peptide nucleic acid (PNA)^19^ oscillate as a function of the length of the molecule. These oscillations were attributed to the coherent conduction through the systems.^17,19^ Hence, we explored the efficiency of the ORR reaction as a function of the length of the DNA molecules adsorbed as monolayers on the gold electrode. Five DNA double strands of various lengths were studied, containing 20, 30, 40, 50, and 70 base pairs (bp). The sequences of the DNA used are provided in the SI.

As was shown in ref (18), while the current through the DNA is generally reduced as the molecule becomes longer, there are oscillations in the current, and the spin polarization tends to increase as a function of length. When the ORR is investigated, it is evident that while the onset of the potential becomes less negative with the length of the DNA, namely lower barrier for the reaction, for the short DNA sequences (20, 30, 40 bp), it becomes much more negative (higher reaction barrier) for the 50 bp long DNA, and then for the 70 bp the barrier is reduced again (Figures 4A,B). When comparing the current density at a potential of −0.45 V, in the case of the three short DNA it is very similar; however, it drops for the 50 bp long DNA and increases significantly for the 70 bp DNA (Figure 4C). From the results it is evident that the ORR reaction characteristics fluctuate as a function of the length of the DNA adsorbed on the electrode. This contrasts with the continuous change in the reaction parameters for electrodes coated with various thicknesses of polymer.

**Figure 4:**
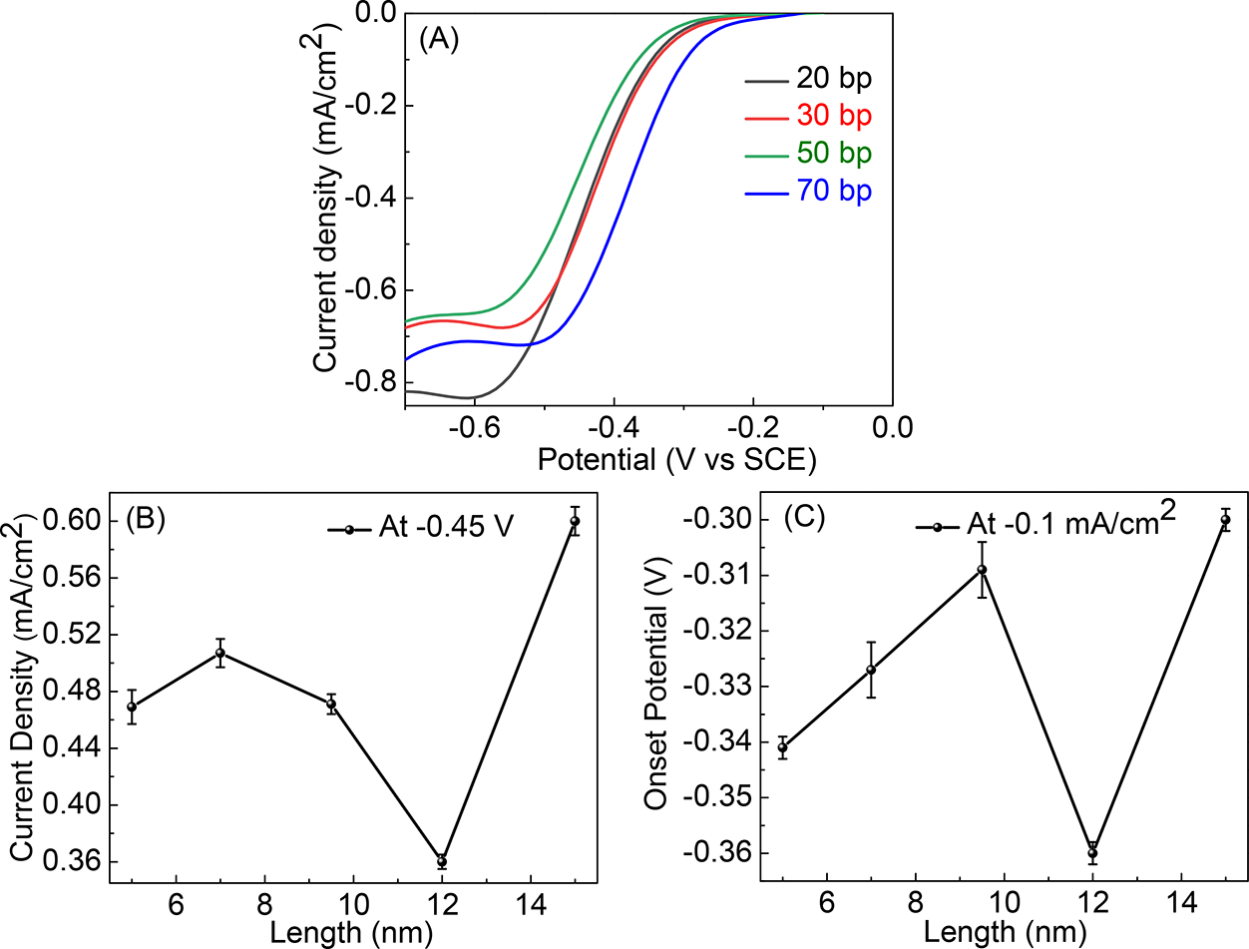
Results from the ORR reaction obtained for working electrode coated with monolayer of double stranded DNA of various lengths. (A) The rising of the CV curve for 20, 30, 50, and 70 bp long DNA. The results from the 40 bp are not presented for clarity, since they overlap with the 20 and 30 bp signal. (B) The current density at a potential of −0.45 V for all 5 lengths of the DNA. (C) The onset potential, defined as the potential for which the current density is 0.1 mA/cm^2^, for the 5 different lengths of DNA.

To compare different biologically related molecules, we performed similar studies with a gold electrode coated with oligopeptides of different lengths (Figure 5). These types of molecules were previously investigated very extensively as spin filters. Specifically, here we investigated short, SHCH_2_CH_2_CO-{Ala-Aib}_5_-COOH, and long (Ala)_4_-Lys-(Ala)_4_-Lys-(Ala)_4_-Lys-(Ala)_4_-Lys-(Ala)_4_-Lys-Ala-COCH_2_CH_2_SH oligopeptides (Al5 and Al36, respectively), where Ala stands for alanine, Aib stands for 2-aminoisobutyric acid, and Lys stands for lysine. These oligopeptides are adsorbed as α-helices.

**Figure 5:**
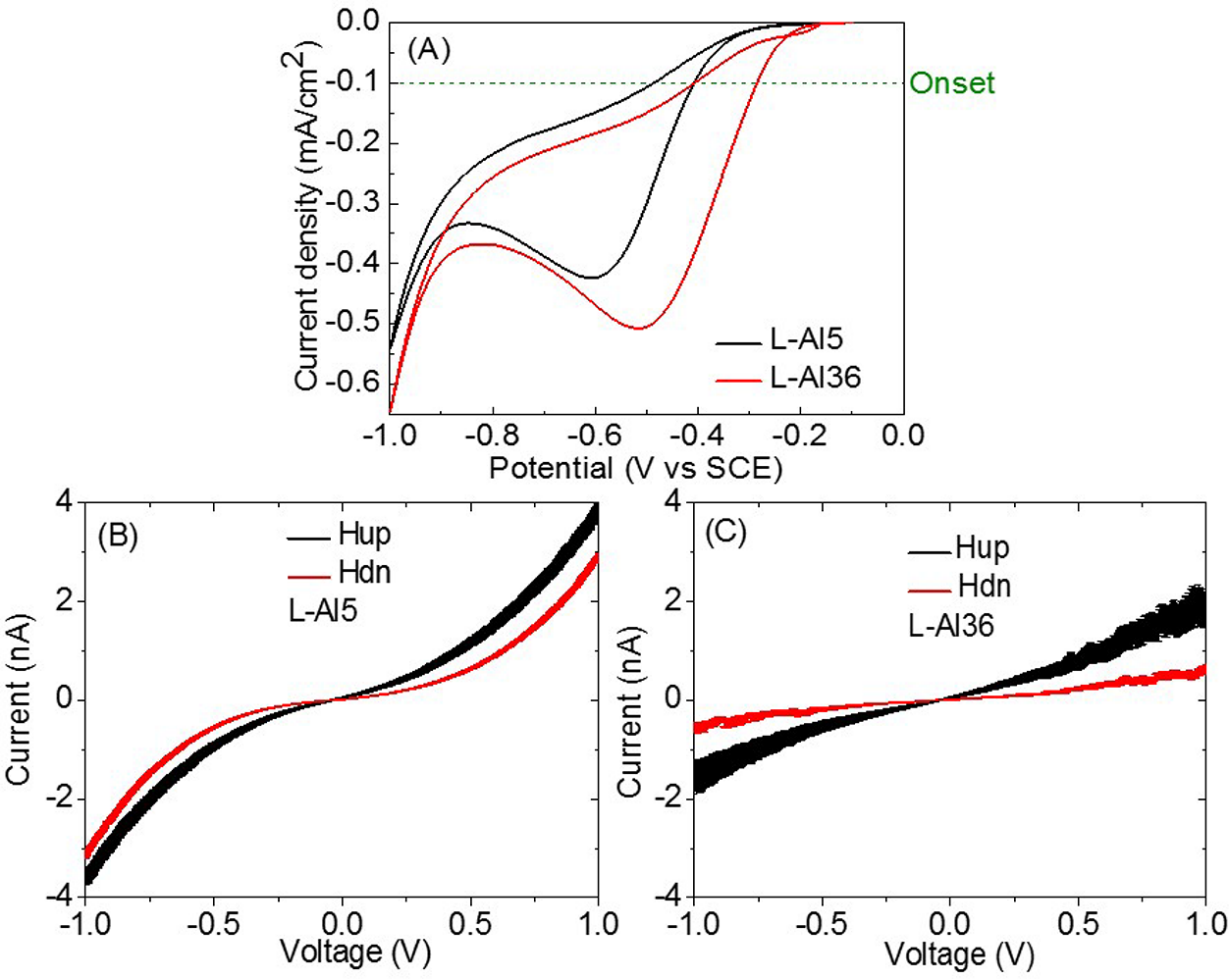
CV plots and the spin-dependent current obtained for electrodes coated with monolayers of α-helices of oligopeptides. (A) The electrochemical CV curves for oligopeptides of two lengths. For the longer oligomer, the peak in the CV shifts to a lower potential. (B,C) The current versus voltage (IV) curves measured for the two oligopeptide lengths. The spin polarization (SP) values measured are shown in the figures. The SP value does not depend on the voltage, once the voltage is higher than about 0.5 V.

In the case of the oligopeptides, as was observed before,^8^ the peak of the current wave is shifted to a lower potential with increasing length as is also observed in the onset potentials. This indicates that the reaction is significantly more efficient with longer oligopeptides. Note that despite the fact that the current is higher for the shorter oligopeptide, the ORR reaction has a lower rate, as indicated by the peak in the CV curve. The results in Figures 4 and 5 indicate that besides the spin polarization and the current, there is another parameter that controls the ORR reaction.

It is known that in the ORR there are two separate channels for two different products that can take place. One is the formation of water, which is the lower energy path, and the second is the formation of hydrogen peroxide, which is the high energy path. The results in Figures 2 and 3 and Table 1 indicate that there is correlation between the peak potential, the threshold potential, and the amount of hydrogen peroxide produced. More hydrogen peroxide is formed when the potentials are higher. Note that the reaction involves the transfer of two pairs of electrons. Clearly, as was shown before, the correlation between the two spin directions in those electrons enhances the reaction and eliminates to a large extent the formation of hydrogen peroxide. However, as presented above, there must be another factor that affects the reaction to rationalize the results presented.

The results raise several dilemmas:

1. Why in the case of a polymer coated electrode does the reaction efficiency drop abruptly for film thickness above 6 nm, while the current decreases monotonically?
2. Why for electrodes coated with different lengths of DNA, does the reaction efficiency fluctuate as a function of the DNA length?
3. Why for DNA and oligopeptides coated electrodes, having the same spin polarization, is the reaction more efficient in the case of oligopeptides?

In the ORR two pairs of electrons are transferred. An intriguing question is whether the electrons in each pair interact with each other and form a coupled pair that is probably coherent or whether the two electrons can be viewed as separate entities. The experimental results seem to indicate the importance of some relationship among the electrons. Calculations were performed to verify the possibility that this relationship relates to coherency.

In the calculations, the ratio between the rates of the single- and two-electron processes was calculated when adding two electrons to a diatomic molecule, which is initially in the spin triplet state. The current for the two-electron process was calculated by assuming that the two electrons are in a coherent state. The details of the model and the calculations are given in the Methods section. The addition of electrons to the molecule is associated with the charge current, *J*_c_, between the electrode and the molecule. In this sense, we account for the physics of the open system comprising the diatomic molecule in contact with an electron reservoir. Specifically, we employed the method used in ref (20), in which the current, *J*_e_, involving single-electron processes, is already provided. At the same level of approximation, we derive the current, *J*_2e_, associated with two-electron processes (see the Methods section). The total charge current is effectively given by *J*_c_ = *J*_e_ + *J*_2e_.

The single- and two-electron processes are calculated when the overlap between the substrate and the molecule is parametrized in terms of the coupling strength for single- and two-electron processes between the substrate and the molecule, Γ and α, respectively. We then simulate the current flux between the substrate and molecule as a function of the ratio α/Γ. An example is shown in Figure 6. Three specific observations can be made here: A. The pertinent single electron current is constant for nearly all changes in the coupling strength, α, of the two-electron additions. B. The two-electron process is very sensitive to α. C. Interestingly, the model, including the spin orbit coupling, did not significantly change the results.

**Figure 6:**
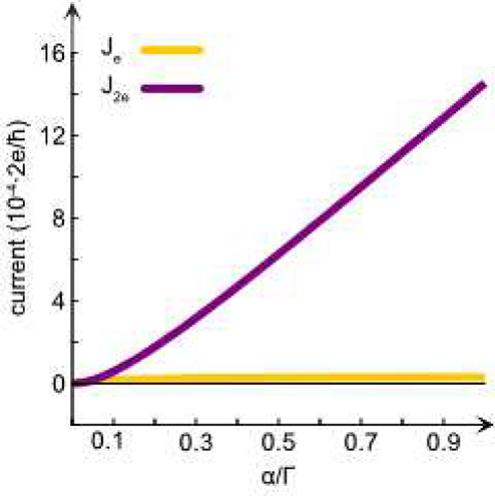
Process-resolved currents between the substrate and the molecule. Bold orange: the total current involving single-electron transitions. Bold purple: the total current involving two-electron transitions for a different ratio of coupling strength between the substrate and the molecule for single- and two-electron processes, Γ and α, respectively. The diatomic molecule is described as two single electron levels, ϵ_*m*_, which are coupled via tunneling, *t*. Moreover, the electrons experience on-site, *U*, and intersite, *U*^′^, Coulomb repulsion, as well as direct exchange, *J*. Here, we used (units: eV) ϵ_*m*_ = −4, *t* = 0.21, *U* = 1.8, *U*^′^ = *U*/4, *J* = −(*U* – *U*^′^)/2, Γ = 0.001, and temperature *T* = 300 K. *J*_T_ is the triplet currents, whereas *J*_S_ is the singlet currents to the electron state.

The results from the simulations summarized in Figure 6 indicate a few important aspects of the possible chemistry related to the oxygen reduction process. When the two electrons interact weakly with the substrate, the reduction by two sequential electrons becomes inefficient. For stronger interactions, the two-electron processes are substantially more efficient than the two sequential single-electron processes.

The model calculations assume two possibilities: either each of the electrons in the pair is independent (a one plus one electron transfer process) or the two electrons are coherently coupled; namely, there is a phase relationship between them. In the second case, the reaction efficiency is larger than those of the two “independent” electrons that have the same spin. This conclusion, based on a quantum mechanical model, is consistent with the classical theoretical model presented in ref (10). It is important to understand that if the electrons do not interact coherently with the oxygen, once the first electron reacts, a Coulomb repulsion exists that increases the barrier for the reaction with the second electron.

The coherent model, presented schematically in Figure 7, is consistent with the thickness dependence of the reaction rate shown in Figures 1 and 2. As the thickness of the chiral polymer increases, the spin polarization of the electrons increases; however, the electrons undergo more collisions and, hence, lose their relative coherency and coupling. As a result, they reduce oxygen as independent single electrons. Therefore, for a polymer thickness exceeding about 7 nm, the ORR rate decreases, and the hydrogen peroxide production increases.

**Figure 7:**
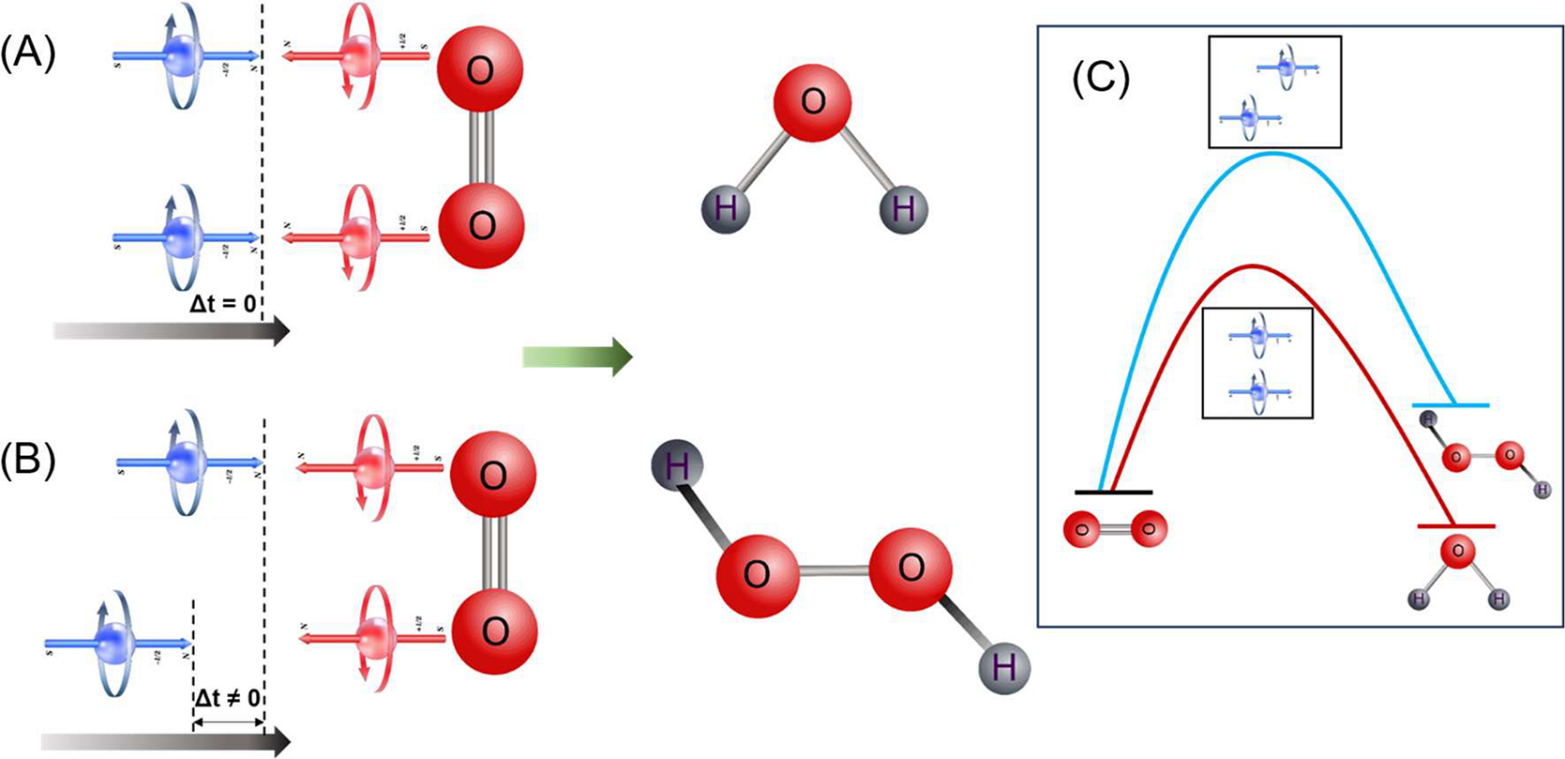
Schematic presentation of the model for ORR with its two paths. (A) When the two electrons in each pair interact and form a coherent pair, the reaction is efficient and water is produced. If the two electrons are not coherent (presented as some delay in their approach), then the reaction product is hydrogen peroxide. (C) The production of hydrogen peroxide is the high energy path that results from the two electrons not inserted coherently into the oxygen.

The fluctuations observed in the efficiency of the reaction for electrodes coated with different lengths of DNA can be explained by the coherent properties of two electrons in the reaction, which result in interferences, which causes fluctuations in the reaction rate. This is consistent with the current fluctuations as a function of DNA length, reported before.^17−19^

If we compare the results obtained with DNA and oligopeptides, we realize that although the spin polarization is about the same for the two types of molecules, the DNA layer is thicker, and indeed one finds that the reaction is less efficient for DNA than for the oligopeptides (Figure 4). The comparison between the two types of molecules also indicates that for DNA the longer oligomer shows a slightly less efficient reaction, despite the spin polarization being higher. However, for oligopeptides, the reaction efficiency significantly increases with longer oligomers. It is important to appreciate that while the longer oligopeptide is only 6 nm long, in the case of DNA even a short molecule with about 40 bp has a length of 10 nm. Hence, the better reaction efficiency is clearly related to the fact that for the same spin polarization, the oligopeptides are much shorter and hence the coherency can be maintained. This finding is especially interesting, since in proteins, α-helices oligomers are one of the most important structural motifs, and electron transport through them is an important feature in Biology; it was found to depend on the spins.^21,22^ Hence, one can conclude that the α-helix structure is both an efficient spin filter and maintains the electron’s coherency.

In the present work, we found a correlation between the production of hydrogen peroxide and the number of collisions the electrons encountered before interacting with oxygen. The hydrogen peroxide production indicates a reduction in the efficiency of the two-electron transfer process in ORR. We suggest the possibility for the coherent relation being important for the efficiency of the reaction. Since collisions may reduce the coherent relationship between the electrons, this further supports the possibility that coherence may be important.

The concept of applying the coherent properties of electrons to control chemical reactions has fascinated the chemical community for several decades. Typically, the aim is to induce coherent properties by using the coherent properties of light, which excite the molecules to a specific coherent superposition of electronic states.^23,24^ Here we presented another concept in which the properties of a pair of electrons transmitted to oxygen are controlled by their path before interacting with the oxygen, and these properties can be probed by the potential barrier of the reaction.

Because the oxygen molecule has a triplet ground state, both its formation in the oxygen evolution reaction (OER) and its decomposition in the oxygen reduction reaction (ORR) are involved in the transmission of pairs of electrons. Since multiple electron redox processes are common in Chemistry and especially in Biology, the present work suggests that the room temperature coherent effects could be of importance. The ORR can serve for establishing a relationship between electrons in multielectron currents. An important step to strengthen the proposed model will be to determine the extent of the entanglement and the phase relationships of the two electrons. The possible role of coherent properties of electrons in chemical processes related to Biology was suggested before.^25^ The present work serves as another encouragement to look for evidence for such a mechanism.

## Supporting Information Available

Additional details regarding methods and materials including polymer coated substrate preparation, electrochemical measurements, magnetic conducting-probe atomic force microscopy (mc-AFM), Theoretical calculations (PDF)
