# Supplementary material for: Does Coherence Affect the Multielectron Oxygen Reduction Reaction?
Source: J Phys Chem Lett. 2023 Oct 12;14(42):9377–84. doi: 10.1021/acs.jpclett.3c02594 (PMC10614294; doi:10.1021/acs.jpclett.3c02594)
Supplement: Supplementary file 1 — jz3c02594_si_001.pdf [file jz3c02594_si_001.pdf]

Supporting Information
Does Coherence Affect the Multi-electron Oxygen Reduction Reaction?

Anu Gupta[1a], Anil Kumar[1a], Deb Kumar Bhowmick[1a], Claudio Fontanesi[2], Yossi Paltiel[3*], Jonas Fransson[4*], and Ron Naaman[1*]

1) Dept. of Chemical and Biological Physics, Weizmann Institute of Science, Rehovot 7610001, Israel
2) Dip. di Ingegneria, DIEF, MO26, University of Modena; 41125 Modena, Italy.
3) Department of Applied Physics and Center for Nanoscience and Nanotechnology, The Hebrew University, Jerusalem 9190401, Israel
4) Department of Physics and Astronomy, Uppsala University, Uppsala 752 36, Sweden.

*Yossi Paltiel, Jonas Fransson, Ron Naaman

Email: paltiel@mail.huji.ac.il, jonas.fransson@physics.uu.se, ron.naaman@weizmann.ac.il

a) The three first authors contributed equally.

This PDF file includes:

Methods and Materials
Supporting text
Figures S1 to S4
SI References

Methods and Materials

Materials:

The substrates for all experiments were prepared using the thermal evaporation deposition technique. A 60 nm layer of Ni layer is sputtered, followed by an 8 nm layer of an Au layer on a Si (100) wafer, with an 8 nm Ti layer as an adhesion layer.

Polymer-coated substrate preparation:

Polymer-coated substrates were prepared via electropolymerization on gold-coated nickel substrates as described in ref. 11. The deposited multilayer surfaces were cleaned by immersing them first in boiling acetone and then in ethanol for 10 minutes. All electrochemical measurements were carried out using a PalmSens4 potentiostat. All experiments were carried out in a three-electrode configuration where $Ag/AgCl_{sat}$ with a saturated KCl solution and a platinum wire, nickel-gold–coated surfaces as a reference, counter electrodes, and working electrodes were used. Polymers were deposited by the chronoamperometry method at a potential of 1.2 V from a solution of 0.25 M 2-vinyl pyridine and 0.05 M NH_4ClO_4 in a 9:1 water-methanol mixture at pH 4.8. Polymerizations were carried out at different times in the presence of the north magnetic field.

Electrochemical measurements:

The electrochemical measurements were performed using a three-electrode closed cell configuration. An Hg/Hg_2Cl_2/saturated KCl (saturated calomel electrode, SCE) and a Pt wire were used as the reference electrode (RE) and the counter electrode (CE), respectively. The working electrode was fixed to the bottom of a Teflon cell through an O-ring with an area of 0.76 cm^2. It is important to mention that the working electrode was static during the measurement. The electrochemical data were taken at room temperature on a potentiostat (PalmSens4) electrochemical workstation using PSTrace software.

A 0.1 M KOH solution (pH = 12.6) was used as the electrolyte solution. Before performing each experiment, the electrolyte was purged with O_2 for 30 min. It is worth mentioning that to maintain the O_2 concentration in the electrolyte solution, the electrochemical cell was closed with only openings for RE, CE, and a needle for purging the gas. During the measurement, the needle for O_2 purging was removed from the solution to maintain a stable

current; however, it remained on top of the solution to avoid a change in O_2 concentration. The oxygen reduction data were taken at room temperature using cyclic voltammetry (CV) at a scan rate of 50 mV/s.

Calculations

The model we set up for the processes is captured in a dimer that may carry anything between none up to four electrons. The dimer is described as two single electron levels, ϵ_m, which are coupled via tunneling, t. Moreover, the electrons experience on-site, U, and inter-site, U', Coulomb repulsion, as well as direct exchange, J. With these parameters, we define the energy space for the dimer and, in particular, we are able to control the configuration. The ground state of the dimer is set to the triplet state $|S=1, m_z=0,\pm 1\rangle$. The Hamiltonian for the dimer can be formulated as

$$\mathcal{H}_{dimer} = \sum_m (\epsilon_m n_m + U n_{m\uparrow} n_{m\downarrow}) + (U' - J/2) n_1 n_2 - 2J \boldsymbol{s}_1 \cdot \boldsymbol{s}_2 + t(\psi_1^\dagger \psi_2 + H.c.),$$

where $n_{m\sigma}$, $\sigma = \uparrow, \downarrow$, denotes the operator for the occupation at level m and spin σ, such that $n_m = n_{m\uparrow} + n_{m\downarrow}$, whereas $\boldsymbol{s}_m$ is the operator for the associated electron spin.

We enable electrons to be added to the molecule by coupling the Hamiltonian

$$\mathcal{H}_T = \sum_{\boldsymbol{k}m\sigma} v_{\boldsymbol{k}m\sigma} \psi_{\boldsymbol{k}\sigma}^\dagger \psi_{m\sigma} + \sum_{\boldsymbol{k}m\sigma} \sum_{\boldsymbol{k}'m'\sigma'} w_{\boldsymbol{k}\boldsymbol{k}'mm'\sigma\sigma'} \psi_{\boldsymbol{k}\sigma}^\dagger \psi_{\boldsymbol{k}'\sigma'}^\dagger \psi_{m'\sigma'} \psi_{m\sigma} + \text{H.c.},$$

where the first and second terms account for the single and two-electron additions, respectively. Here, $v_{\boldsymbol{k}m\sigma}$ and $w_{\boldsymbol{k}\boldsymbol{k}'mm'\sigma\sigma'}$ denote the respective tunneling rates, whereas $\psi_{\boldsymbol{k}\sigma}$ ($\psi_{\boldsymbol{k}\sigma}^\dagger$) and $\psi_{m\sigma}$ ($\psi_{m\sigma}^\dagger$) are the electron destruction (creation) operators for the corresponding electron processes in the substrate and molecule. The electrons transferred to the molecule come from a substrate modeled as a simple free electron gas, characterized by the band energy $\epsilon_{\boldsymbol{k}}$, through $\mathcal{H}_{sub} = \sum_{\boldsymbol{k}\sigma} \epsilon_{\boldsymbol{k}} \psi_{\boldsymbol{k}\sigma}^\dagger \psi_{\boldsymbol{k}\sigma}$.

The simulations summarized in Figure 6 indicate a few important aspects of the possible chemistry related to the oxygen reduction process. First, despite that the single-electron processes are not forbidden, it is only when the two-electron overlap between the substrate and molecule, parametrized by α, is much smaller than the corresponding single

electron overlap, parametrized by Γ, that the reduction process becomes inefficient. Indeed, in Figure 5, this regime was found to be below a ratio of about 0.1. Above this number, a critical ratio, the two-electron processes are substantially more efficient and already at unit ratio, and the two-electron processes become about **7 times** as efficient as the single-electron processes in the given range of the ratio α/Γ.

Another aspect, which can be seen in Figure 5, is that the triplet currents (J_{T1}, J_{T2}, J_{T3}) are not equal, whereas the singlet currents (J_{S1}, J_{S2}, J_{S3}) vanish. This is, however, not a contradiction, since the actual triplet configurations occurring in the simulations are mixtures of the states $[|\uparrow\rangle|\downarrow\rangle + |\downarrow\rangle|\uparrow\rangle]/\sqrt{2}$ and $|\sigma\rangle|\sigma\rangle, \sigma = \uparrow, \downarrow$. This, in turn, leads to the conclusion that the currents involving the triplets in the simulations may not be a third of the total.

In the above simulations, any possible spin-orbit interaction has been omitted. This assumption is reasonable whenever the triplet states can be unequivocally defined. However, allowing for a spin-orbit coupling in the transfer between two- and four-electron states, i.e., the processes corresponding to the oxygen reduction reaction, also opens transitions involving two-electron transfer from the singlet states. Therefore, we compared the ratios J_T/J_{2e} and J_S/J_{2e}, where $J_{T(S)}$ denotes the two-electron currents from the triplet (singlet) states to the four-electron state, whereas $J_{2e} = J_T + J_S$ is the total two-electron current between the two and four electron states.

Supporting Information Text

DNA sequences used

20bp dsDNA

CGC TTC GCT TCG CTT CGC TT/3ThioMC3-D/

AAG CGA AGC GAA GCG AAG CG

30bp dsDNA

CGC TTC GCT TCG CTT CGC TTC GCT TCG CTT/3ThioMC3-D/

AAG CGA AGC GAA GCG AAG CGA AGC GAA GCG

40bp dsDNA

CGC TTC GCT TCG CTT CGC TTC GCT TCG CTT CGC TTC GCT T/3ThioMC3-D/

AAG CGA AGC GAA GCG AAG CGA AGC GAA GCG AAG CGA AGC G

50bp dsDNA

CGC TTC GCT TCG CTT CGC TTC GCT TCG CTT CGC TTC GCT TCG CTT CGC TT/3ThioMC3-D/

AAG CGA AGC GAA GCG AAG CGA AGC GAA GCG AAG CGA AGC GAA GCG AAG CG

70bp dsDNA

TAC TCT ACC TTC TCA AGA ATC GGC ATT AGC TCA ACT GTC AAC TCC TCT ACC TTC TCA AGA ATC/3ThioMC3-D/

AAA TGC CGA TTC TTG AGA AGG TAG AGG AGT TGA CAG TTG AGC TAA TGC CGA TTC TTG AGA AGG TAG AGT A

Hydrogen peroxide quantification

The formation of hydrogen peroxide was confirmed in a colorimetric test titration experiment of solutions used for electrochemical ORR. o-tolidine was used as a redox indicator. The electrochemistry measurements were performed in chronoamperometry mode to quantify hydrogen peroxide as a function of the working electrode polymer thickness. Chronoamperometries were run by applying a constant potential of -0.5 V for 30 minutes in an O_2-saturated 0.1 M KOH solution (Figure S1). During the chronoamperometry, the needle for O_2 purging was kept at the top of the solution to avoid

current fluctuations. After chronoamperometry measurements, the KOH solution was removed. The redox indicator o-tolidine needs an acidic medium to react with the KOH solution. Therefore, 1 ml of 1 M HCl was added to 2 ml of 0.1 M KOH solution. Afterwards, in 1 ml of the final solution, 0.2 ml of an o-tolidine 0.94 mM aqueous solution was added and left at rest to react for 30 minutes. After 30 min, the absorbance of the solution was measured (as shown in Figure-S1) using a Varian Cary 50 Bio UV/Visible spectrometer. The yellow solution, featuring an absorption peak at around 436 nm, confirms the formation of H_2O_2 [1].

Atomic force microscopy with a magnetic conducting probe (mc-AFM)
The polymer film, measured via magnetic conductive probe atomic force microscopy (mc-AFM), was prepared electrochemically when the working electrode is magnetized so that its north pole points towards the solution. The magnetic field-dependent current-voltage (*I-V*) characteristics of the prepared samples were determined using a multimodal scanning magnetic probe microscopy (SPM) system equipped with a Beetle Ambient AFM and an electromagnet with an R9 electronic controller (RHK Technology). Voltage spectroscopy for the *I-V* measurements was performed by applying voltage ramps with a non-magnetic Pt tip (DPE-XSC11, μmasch with spring constant 3-5 Nm^{-1}) in contact mode. The representative results of the mc-AFM experiments are shown in Figure-S2.

Current-Voltage (*I-V*) spectra were acquired (with the magnetic field up or down) from several points of the polymer samples; the corresponding average I-V curves are shown in Figure-S2. Each bold red/black curve represents the average of more than 50 I-V curves and representative plots of the average IV curves are depicted in Figure-S3. Clearly, two different onsets of the currents were observed in different magnetic field (up or dn) directions that correspond to the presence of spin polarization. Moreover, the distinct threshold for each spin indicates that no spin flipping occurs during the conduction process. Furthermore, the percentage of spin polarization (SP%) for all samples is calculated using the relation, SP%= $\frac{(I_{up}-I_{dn})}{(I_{up}+I_{dn})}\times 100$, where I_{up} and I_{dn} represent the current in the up or dn direction, respectively. The estimated percentage of SP% for samples having thicknesses of 4 nm, 7 nm, 15 nm, and 20 nm samples is of the order of 20%±5, 30%±8, 45±6 and 55%±8, respectively (see Figure-S2). The systematic scaling in the values of spin

polarization can be explained in terms of an increase in the electric polarization due to enhancement in the length of the polymer samples. Therefore, charge polarization, accompanied by spin polarization, seems to be the main factor that contributes to the aforementioned behavior of spin polarization. The I-V curves for DNA and the oligopeptide samples are shown in Figures-S4 and S5, respectively.

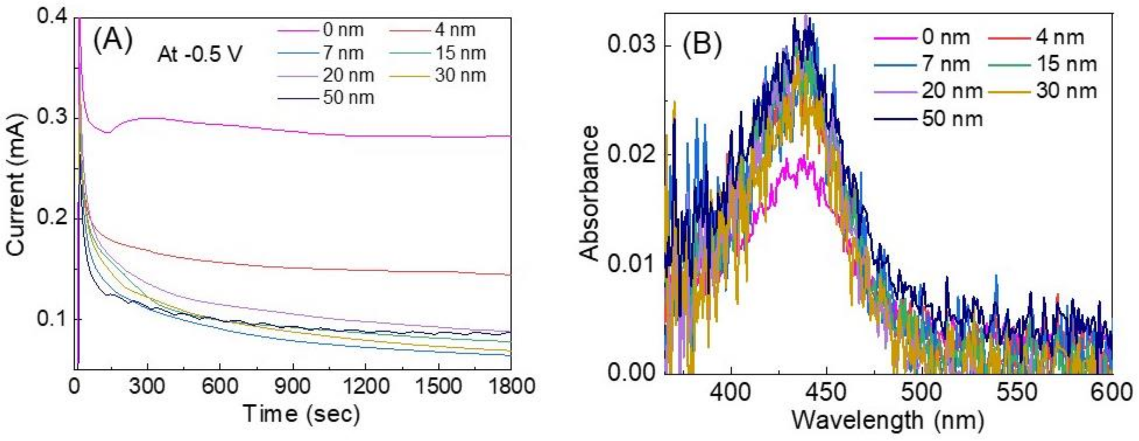

Figure S1: (A) Chronoamperometric measurements were taken at constant potential of -0.5 V for 30 minutes. (B) UV-Vis absorption spectra of o-tolidine taken from the electrochemical cell after chronoamperometry. Different spectra refer to different polymer thicknesses covering the working electrode. The thickness values are indicated in the figure.

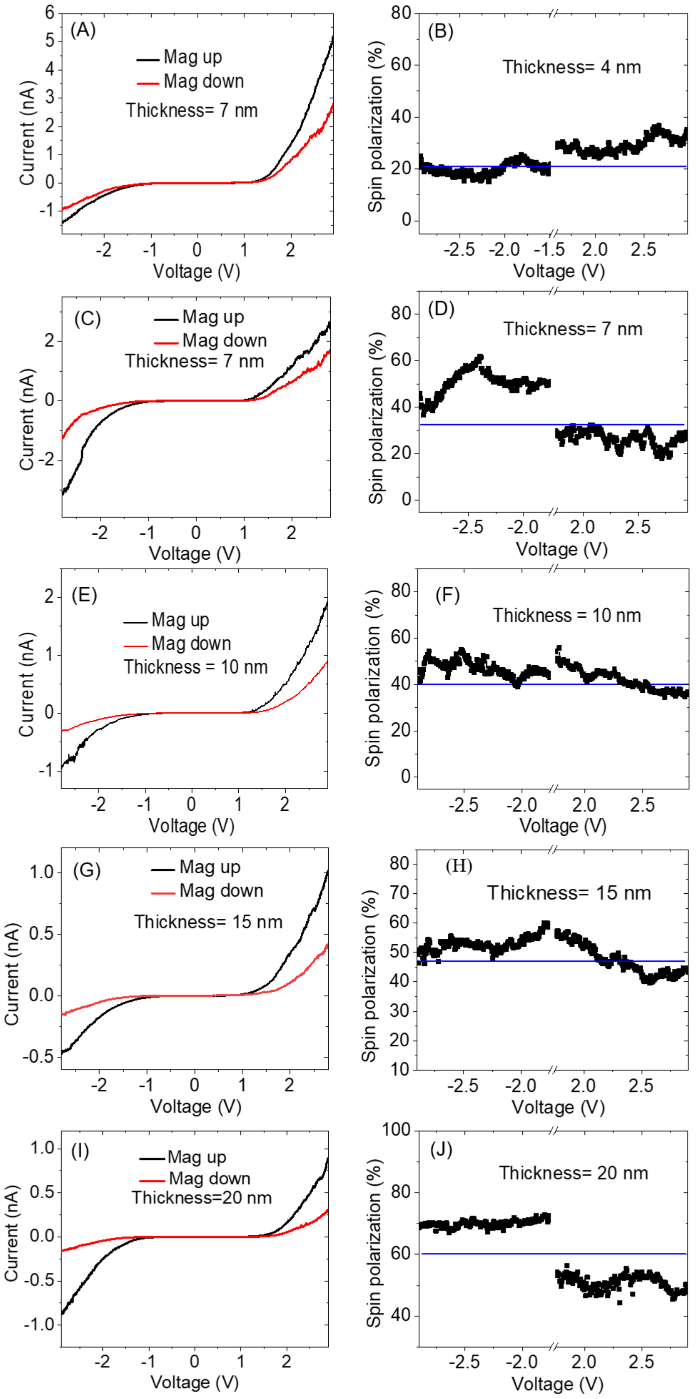

Figure S2. Spin-dependent transport properties were measured with the mc-AFM as a function of the polymer thickness. Left Panels A, C, E, G, and I present the average current versus voltage (I−V) curves recorded for all polymer samples with the magnet north pole pointing down (red) or up (black). Right Panels B, D, F, H, and J represent the corresponding spin polarization of all polymer samples, SP=$\frac{I_{up}-I_{down}}{I_{up}+I_{down}}$ when I_{up} and I_{down} are the current with the magnet north pole pointing up or down, respectively.

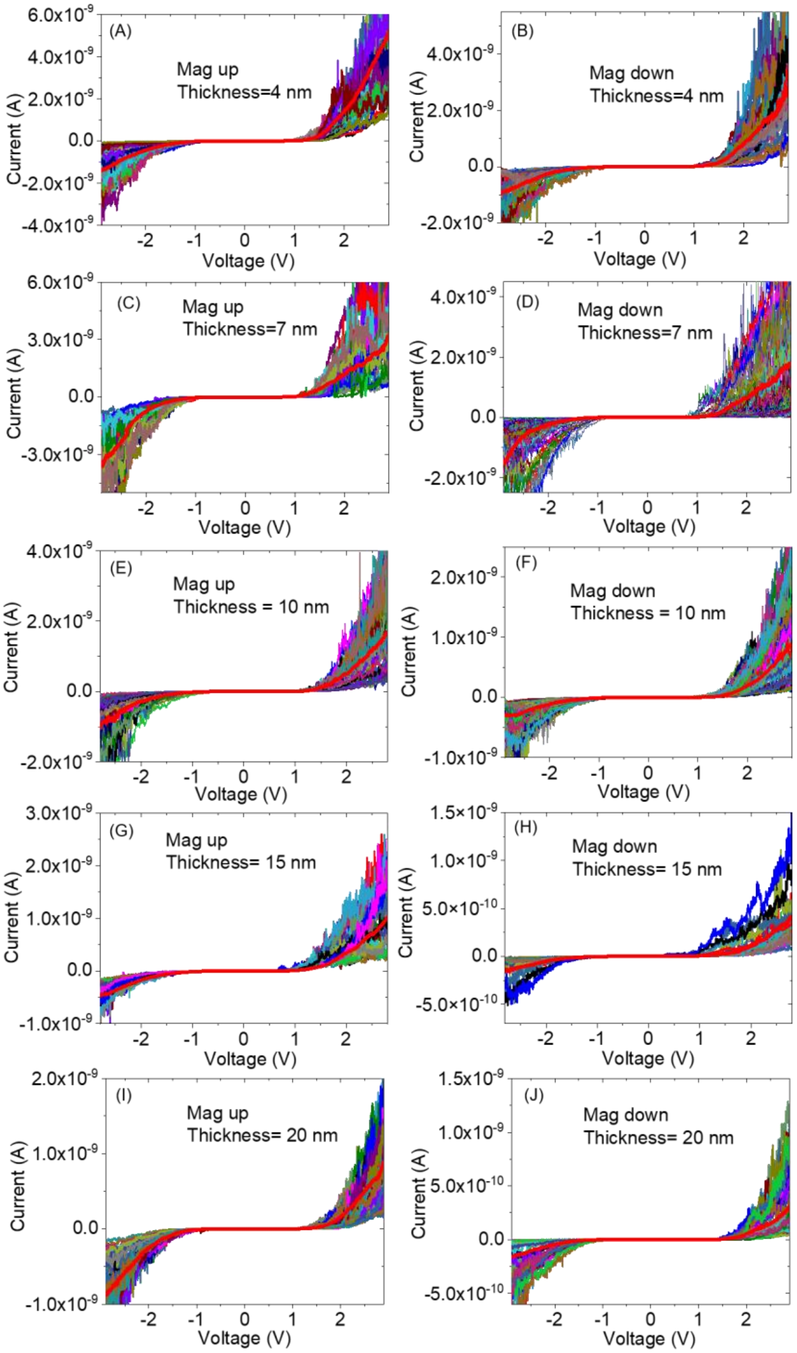

Figure S3: Spin-dependent transport for different thickness polymer samples with the magnet north pole pointing up and down. The left panels A, C, E, G, and I show the spin transport data of different polymer thicknesses with magnet pointing up. The left panels B, D, F, H, and J show the spin transport data of different polymer thicknesses with the magnet pointing down. The bold red curve denotes the average of more than 50 I-V curves.

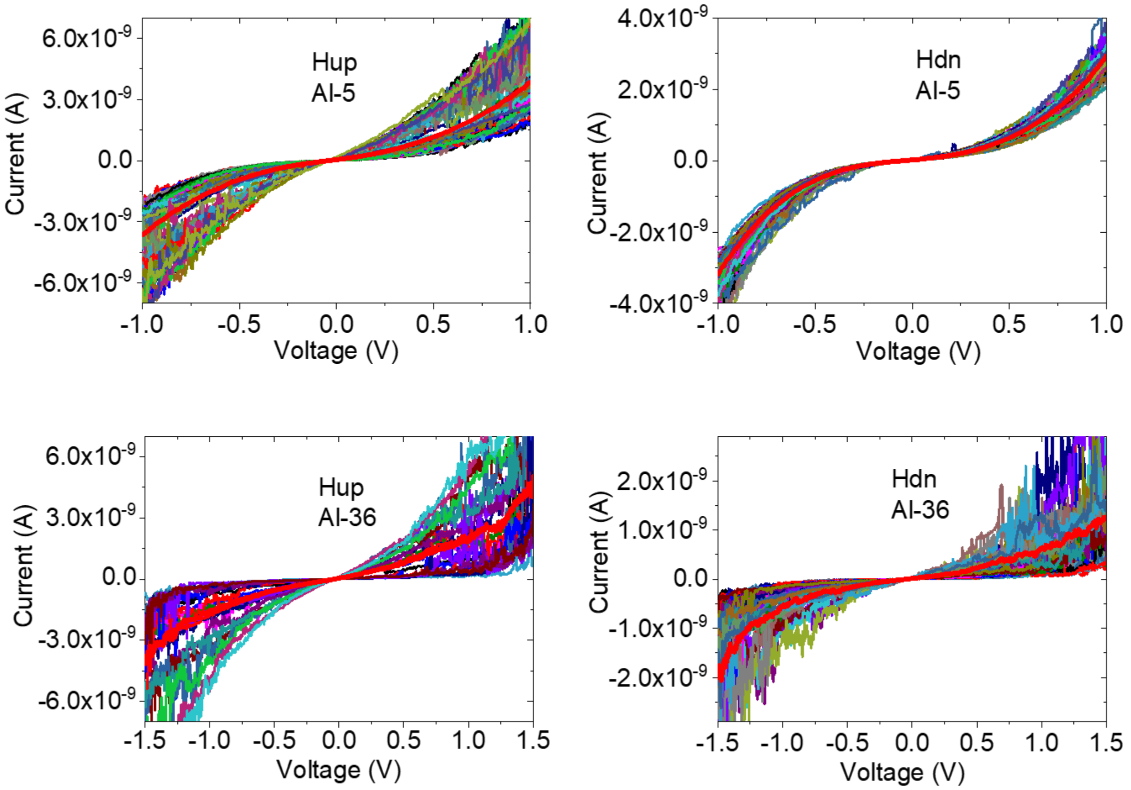

Figure S4: The representative IV curves for different lengths of oligopeptide samples with the magnet north pole pointing up (Panels A and C) and down (Panels B and D). The bold red curve denotes the average for more than 50 I-V curves.

SI References

(1) Hansen, W. N.; Kuwana, T.; Osteryoung, R. A. Observation of Electrode-solution Interface by Means of Internal Reflection Spectrometry. *Anal. Chem.* **1966**, *38*, 1810−1821.
